# Examination of Text Message Plans and Baseline Usage of Families Enrolled in a Text Message Influenza Vaccine Reminder Trial: Survey Study

**DOI:** 10.2196/39576

**Published:** 2023-06-30

**Authors:** Chelsea S Wynn, Alexander G Fiks, Russell Localio, Justine Shults, Ekaterina Nekrasova, Laura P Shone, Alessandra Torres, Miranda Griffith, Rebecca Unger, Leigh Ann Ware, Mary Kate Kelly, Melissa S Stockwell

**Affiliations:** 1 Division of Child and Adolescent Health Department of Pediatrics Columbia University Vagelos College of Physicians and Surgeons New York, NY United States; 2 Department of Pediatrics Clinical Futures & PolicyLab Children's Hospital of Philadelphia Philadelphia, PA United States; 3 Department of Biostatistics, Epidemiology & Informatics University of Pennsylvania Philadelphia, PA United States; 4 Primary Care Research American Academy of Pediatrics Itasca, IL United States; 5 Northwestern Children’s Practice Chicago, IL United States; 6 Building Blocks Pediatrics Pleasanton, TX United States; 7 Department of Population and Family Health Columbia University Mailman School of Public Health New York, NY United States

**Keywords:** influenza vaccine, mHealth, mobile phone, pediatric, primary care, PROS, reminders, text message

## Abstract

**Background:**

Mobile health (mHealth) is quickly expanding as a method of health promotion, but some interventions may not be familiar or comfortable for potential users. SMS text messaging has been investigated as a low-cost, accessible way to provide vaccine reminders. Most (97%) US adults own a cellphone and of those adults most use SMS text messaging. However, understanding patterns of SMS text message plan type and use in diverse primary care populations needs more investigation.

**Objective:**

We sought to use a survey to examine baseline SMS text messaging and data plan patterns among families willing to accept SMS text message vaccine reminders.

**Methods:**

As part of a National Institutes of Health (NIH)–funded national study (Flu2Text) conducted during the 2017-2018 and 2018-2019 influenza seasons, families of children needing a second seasonal influenza vaccine dose were recruited in pediatric primary care offices at the time of their first dose. Practices were from the American Academy of Pediatrics’ (AAP) Pediatric Research in Office Settings (PROS) research network, the Children’s Hospital of Philadelphia, and Columbia University. A survey was administered via telephone (Season 1) or electronically (Season 2) at enrollment. Standardized (adjusted) proportions for SMS text message plan type and texting frequency were calculated using logistic regression that was adjusted for child and caregiver demographics.

**Results:**

Responses were collected from 1439 participants (69% of enrolled). The mean caregiver age was 32 (SD 6) years, and most children (n=1355, 94.2%) were aged 6-23 months. Most (n=1357, 94.3%) families were English-speaking. Most (n=1331, 92.8%) but not all participants had an unlimited SMS text messaging plan and sent or received texts at least once daily (n=1313, 91.5%). SMS text messaging plan type and use at baseline was uniform across most but not all subgroups. However, there were some differences in the study population’s SMS text messaging plan type and usage. Caregivers who wanted Spanish SMS text messages were less likely than those who chose English to have an unlimited SMS text messaging plan (n=61, 86.7% vs n=1270, 94%; risk difference –7.2%, 95% CI –27.1 to –1.8). There were no significant differences in having an unlimited plan associated with child’s race, ethnicity, age, health status, insurance type, or caregiver education level. SMS text messaging use at baseline was not uniform across all subgroups. Nearly three-quarters (n=1030, 71.9%) of participants had received some form of SMS text message from their doctor’s office; most common were appointment reminders (n=1014, 98.4%), prescription (n=300, 29.1%), and laboratory notifications (n=117, 11.4%). Even the majority (n=64, 61.5%) of those who did not have unlimited plans and who texted less than daily (n=72, 59%) reported receipt of these SMS text messages.

**Conclusions:**

In this study, most participants had access to unlimited SMS text messaging plans and texted at least once daily. However, infrequent texting and lack of access to an unlimited SMS text messaging plan did not preclude enrolling to receive SMS text message reminders in pediatric primary care settings.

## Introduction

Mobile health interventions (mHealth) are gaining prominence as cellphone use has become an integral component of societal connectedness. Ninety-seven percent of US adults own a cell phone and coverage rates are high across diverse populations [1]. Many types of mHealth interventions, using a variety of media such as mobile apps [2], SMS text messaging [3], and patient portals [4], have been instrumental in promoting gains for patient users in several critical public health areas such as smoking cessation [5], mental health care [6], diabetes care management [7], and vaccination [8]. However, some mHealth interventions may be outpacing the technological ability and familiarity of their users, especially those who were introduced to personal electronic use in adulthood. Mobile phone applications, while providing many resources for users, require download and at times multi-step use instructions that may take away from the ease of use. They also require a data plan or consistent access to Wi-Fi, which differ along demographic characteristics such as age, sex, health, and income [9]. Given the complexity of certain mHealth apps, the simplicity of SMS text messaging interventions in reaching varied populations improves accessibility.

Of the 97% of US adults who own cell phones [1], most cell phone owners use SMS text messaging [10]. Unlike app-based interventions, SMS text message capability is standard on nearly all mobile phones [11,12]. This removes a barrier faced by those who may have difficulty or hesitancy toward using mHealth interventions that require a download onto their phones [13]. SMS text messages may be more likely to reach the intended recipient than an autodialer call to a landline [14]. Cell phone numbers may also be more constant than home addresses or landline numbers [15]. Even when families move, they can keep the same cell phone number [15]; however, smartphone owners making <US $30,000 annually are at increased risk of having discontinuous service and changing phone numbers, which may disproportionally affect those from underserved populations [10,16]. SMS text messaging has also been investigated as a low-cost, accessible way to deliver health messages in the pediatric setting for many areas of health promotion, including vaccine reminders [14,17-22].

While SMS text message use is becoming more widespread, it is important to assess user interaction and familiarity with this modality when considering implementation to promote health. For example, SMS text messaging plans that require users to pay-per-text may present a barrier. Also, if cell phone owners use SMS text messages infrequently or never, this could limit the impact of SMS text messages on health behaviors, potentially contributing to inequities. However, little is known about whether lower texting frequency or SMS text message plan limitations impact willingness to receive SMS text message reminders, particularly within the pediatric setting. We hypothesized that given the ease of use and accessibility of SMS text messages, caregivers and families would show widespread use of SMS text messages, even with differences in plan and usage. In this study, we asked families who had already agreed to receive SMS text message reminders, about their SMS text messaging plans and prior patterns of SMS text message use.

## Methods

### Study Design, Setting, and Population

Funded by the National Institute of Health (NIH R01HD086045), the Flu2Text randomized control trial was conducted during the 2017-2018 and 2018-2019 influenza seasons to investigate whether SMS text message reminders can impact the effectiveness and timeliness of receipt of the second dose of influenza vaccine for those who need 2 doses in a season [23]. Of the 50 practices in this study, 46 were from the Pediatric Research in Office Settings (PROS) Network, the pediatric primary-care practice-based research network of the American Academy of Pediatrics (AAP), and the remaining 4 were affiliated with the Children’s Hospital of Philadelphia (CHOP) and Columbia University, respectively. Practices were located throughout the United States (Northeast [28%], South [36%], Midwest [16%], and West [20%]).

A convenience sample of caregivers of children needing a second dose of influenza vaccine in either season were recruited in primary care offices at the time of their first dose. The eligibility criteria included communicating in English or Spanish and having a cell phone with SMS text messaging capabilities. During the 2017-2018 influenza season, caregivers gave consent and completed the demographic survey by phone with contract research staff. During the 2018-2019 influenza season, caregivers gave consent verbally at their child’s primary care office and completed the demographic survey via a web-based link embedded in an SMS text message approximately 1 to 3 days after enrollment.

The survey included questions on baseline SMS text message use and experiences with medical-related SMS text messages, and was based on the previous surveys used by the Columbia study team [24].

All survey materials were completed in either English or Spanish, based on the caregiver’s SMS text message language preference.

### Ethics Approval

Institutional Review Boards at Columbia University (AAAR4101), the Children’s Hospital Of Philadelphia (17-013735), the American Academy of Pediatrics (17 ST 01) approved this study with a waiver of written consent, and the University of South Carolina relied on the American Academy of Pediatrics Institutional Review Board.

### Outcomes

Our primary outcomes were caregiver-reported: (1) SMS text message plan type and (2) frequency of SMS text message use. Caregivers were asked “What type of text message plan do you currently have?” Possible responses included limited versus unlimited number of SMS text messages per month. Caregivers were also asked “How often do you send and/or receive text messages?” Response categories included at least once a day, at least once a week but not every day, at least once a month but not every week, occasionally but not every month, and never. For the analysis, these categories were collapsed into “at least once a day” versus “less than once a day.”

A secondary outcome was the caregiver’s previous experience with SMS text messages from a doctor or medical office. Specifically, caregivers were asked “Have you ever received a text message from a doctor or their office?” Those who responded “Yes” were prompted to provide the nature of prior messages that they had experienced. Response categories included: appointment reminders, vaccine reminders, notification of laboratory results, notification of school forms being ready, prescription notification, and other.

### Variables

Demographic variables asked of participants were child ethnicity (non-Hispanic or non-Latino and Hispanic or Latino), child race (White, Black, Asian, Native Hawaiian or Pacific Islander, and American Indian or Alaskan Native), child age (6-23 months or ≥2 years), child health (excellent, very good, good, fair, and poor), caregiver’s relationship to the child (mother, father, grandparent, and other), caregiver’s age, caregiver’s preferred language for SMS text messaging (English and Spanish), and child insurance type (commercial, public insurance, Tricare, or uninsured).

For the race variable, we collapsed those who reported having children who were Native Hawaiian or Pacific Islander, American Indian or Alaskan Native, or multiple race categories into an “Other” group due to lower enrollment numbers. Similarly, due to the small number of uninsured participants, those reported as being publicly insured or uninsured were combined into one category for the insurance variable. Those who reported having Tricare for insurance were collapsed into having commercial insurance.

### Analyses

We performed separate multivariable logistic regression analyses to calculate standardized adjusted proportions for the primary outcomes of SMS text message plan type and SMS text messaging frequency, adjusted for the child’s and caregiver’s demographic characteristics described above.

We used the logistic regression models to estimate standardized (adjusted) values of the outcomes by predictive margins. We used bootstrapping to calculate 95% CIs for the risk differences of the standardized and adjusted proportions [25]. All analyses were conducted using Stata version 15.1 [26].

## Results

A total of 2086 parent-child dyads (257 from 2017 to 2018, 1829 from 2018 to 2019) were enrolled. Most (n=1439, 69%) completed the demographic survey. The survey completion rates in the 2017-2018 and 2018-2019 seasons were 100% and 64.6%, respectively. Mean caregiver age was 32 years (SD 6 years), and most children (n=1355, 94.2%) were 6 to 23 months old. Most caregivers (n=1357, 94.3%) preferred English for studying SMS text messages (Table 1).

**Table 1:** Demographic characteristics and SMS text message experiences of the study sample.

Characteristics		Caregivers (N=1439), n (%)
**Child ethnicity**		
	Not Hispanic or Latino	1184 (82.3)
	Hispanic or Latino	255 (17.7)
**Child race^a^**		
	White	906 (64.7)
	Black	274 (19.6)
	Asian	123 (8.8)
	Other^b^	97 (6.9)
**Child age**		
	6-23 months	1355 (94.2)
	2-8 years old	84 (5.8)
**Child health^a^**		
	Excellent	984 (68.6)
	Very good	353 (24.6)
	Good, fair, or poor	97 (6.76)
**Child insurance type^a^**		
	Commercial insurance	907 (63.1)
	Public insurance or uninsured^c^	531 (36.9)
**Caregiver relation to child^a^**		
	Mother	1277 (88.8)
	Father, grandparent, or other	161 (11.2)
**Caregiver age^a^**		
	<30 years	484 (34.6)
	30-34 years	502 (35.9)
	35-39 years	308 (22)
	>40 years	106 (7.6)
**Caregiver education^a^**		
	Masters or doctorate	338 (23.5)
	Associates or bachelors	522 (36.3)
	Vocational school or some college	257 (17.9)
	High school or less	320 (22.3)
**Preferred language for SMS text messages**		
	English	1357 (94.3)
	Spanish	82 (5.7)
**Texting plan type^a^**		
	Limited	104 (7.2)
	Unlimited	1331 (92.8)
**Texting frequency^a^**		
	Send or receive texting everyday	1313 (91.5)
	Send or receive texting<every day or never	122 (8.5)
**Influenza season**		
	2017-2018	257 (17.9)
	2018-2019	1182 (82.1)

^a^Not all 1439 survey participants answered this item.

^b^The children, who were reported as being Native Hawaiian or Other Pacific Islander (n=5), American Indian or Alaska Native (n=9) or were more than one race (n=83), were collapsed into one “Other” category.

^c^Across both seasons there were few uninsured survey participants (n=11); for analysis this category was combined with the publicly insured participants.

We had limited demographic data (gender, age, insurance, and SMS text message preference) available on nonresponders. On chi-square, there were no significant demographic differences among survey responders versus nonresponders for child gender (female vs male). A greater proportion of children of survey completers were 6-23 months old (n=1355, 94.2%) versus 89% (n=576) of children of noncompleters (*P*<.001). Children of survey completers were more likely to have commercial insurance compared with children of noncompleters (n=907, 63.1% vs n=230, 35.6%) (*P*<.001), and to request English text messages rather than Spanish (n=1357, 94.3% vs n=573, 88.6%) (*P*<.001).

Most survey participants reported that they had an unlimited texting plan (n=1331, 92.8%) and texted daily (n=1313, 91.5%). However, there were some differences in the study population’s SMS text message plan type and usage. Caregivers who wanted Spanish SMS text messages were less likely than those who chose English to have an unlimited SMS text messaging plan (n=61, 86.7% vs n=1270, 94%) (risk difference 7.2%, 95% CI [27.1 to 1.8]). There were no significant differences in having an unlimited plan associated with child’s race, ethnicity, age, health status, insurance type, or caregiver’s education level. SMS text messaging use at baseline was not uniform across all subgroups (Table 2).

Nearly three-quarters (n=1030, 71.9%) of participants had previously received some form of SMS text message from a doctor. Nonmothering caregivers were found to be less likely to have received an SMS text message from a doctor (n=96, 58.8%) than caregivers who are mothers (n=933, 73.5%) (risk difference14.7%, 95% CI [23.7 to 5.9]). Additionally, caregivers with a high school education or less were less likely to have experienced receiving an SMS text message from a doctor (n=210, 66.7%) than caregivers with a master’s degree or higher (n=260, 77.7%) (risk difference11%, CI 95% [19.6 to 2.3]). Older caregivers >40 years of age were more likely to have received an SMS text message from a doctor than caregivers younger than 30 years old (n=80, 79.2% vs n=328, 67.8%, respectively) (risk difference 11.4%, 95% CI [0.2 to 21.2]) (Table 3). Of all participants, 98.4% (n=1014) reported receiving appointment reminders, 29.1% (n=300) prescription notifications, 11.4% (n=117) laboratory notifications, 11.3% (n=116) vaccine reminders, and 6.2% (n=64) reminders about school forms. Even the majority of those who did not have unlimited plans (n=64, 61.5%) and those who texted less than daily (n=72, 59%) still reported having received SMS text messages from a doctor’s office at some point in the past.

**Table 2:** Relationship between demographic factors and caregiver-reported SMS text messaging plan type and usage.

		SMS text messaging plan type			SMS text messaging usage frequency		
		Adjusted, n (%)		Risk difference (95% CI)	Adjusted, n (%)		Risk difference (95% CI)
		Unlimited texts	Limited texts		Send or receive daily	Send or receive less than daily	
**Child ethnicity**							
	Not Hispanic or Latino	1109 (93.8)	72 (6.2)	Reference	1098 (92.5)	83 (7.5)	Reference
	Hispanic or Latino	222 (92.2)	32 (7.8)	1.6 (6.4 to 2.5)	215 (88.5)	39 (11.5)	4 (8.4 to 0.5)
**Child race**							
	White	858 (94.4)	46 (5.6)	Reference	868 (95)	38 (5)	Reference
	Black	256 (94.7)	18 (5.3)	0.3 (2.7 to 4)	244 (88.9)	30 (11.1)	6.1 (9.8 to 1.9)
	Asian	103 (83.2)	19 (16.8)	11.2 (21.5 to 0.6)	90 (68.4)	30 (31.6)	26.6 (39.8, 11.8)
	Other	82 (92.8)	15 (7.2)	1.6 (6.6 to 3.9)	80 (94.1)	16 (5.9)	0.9 (8.5 to 2.7)
**Child age**							
	6-23 months	1256 (93.6)	95 (6.4)	Reference	1240 (91.9)	112 (8.1)	Reference
	2-8 years	75 (90.2)	9 (9.8)	3.4 (11.6 to 3.2)	73 (88.9)	10 (11.1)	3 (9.8 to 2.5)
**Child health**							
	Excellent	916 (93.8)	65 (6.2)	Reference	898 (91.5)	84 (8.6)	Reference
	Very good	328 (93.8)	25 (6.2)	0.1 (2.7 to 2.2)	332 (93.8)	21 (6.2)	2.3 (0.6 to 5.2)
	Good, fair, or poor	83 (89.1)	13 (10.9)	4.8 (9.8 to 0.7)	79 (88.3)	17 (11.7)	3.2 (7.9 to 1.5)
**Child insurance type**							
	Commercial insurance	853 (93.8)	52 (6.2)	Reference	847 (92)	58 (8)	Reference
	Public insurance or uninsured	477 (92.8)	52 (7.2)	1 (4.5 to 2.2)	465 (91.4)	64 (8.6)	0.7 (4.5 to 2.8)
**Caregiver relation to child**							
	Mother	1185 (93.4)	89 (6.6)	Reference	1167 (91.6)	108 (8.4)	Reference
	Father, grandparent, or other	145 (93.5)	15 (6.4)	0.1 (4.1 to 4.2)	145 (92.4)	14 (7.6)	0.8 (3.5 to 5.4)
**Caregiver age**							
	<30 years	458 (95)	26 (5)	Reference	443 (92.3)	40 (7.7)	Reference
	30-34 years	467 (92.8)	34 (7.2)	2.1 (5.4 to 1)	467 (92.4)	34 (7.6)	0.1 (3.4 to 3.7)
	35-39 years	284 (92.9)	22 (7.1)	2.1 (5.7 to 2)	272 (89.6)	34 (10.4)	2.7 (7.4 to 1.7)
	>40 years	92 (90.3)	13 (9.7)	4.7 (12.4 to 0.7)	96 (92.1)	10 (7.9)	0.3 (5.7 to 5.2)
**Caregiver education**							
	Masters or doctorate	317 (94.7)	21 (5.3)	Reference	316 (94.8)	21 (5.2)	Reference
	Associates or bachelors	491 (93.7)	29 (6.3)	1 (3.6 to 3.2)	488 (92)	33 (8)	2.8 (5.6 to 0.6)
	Vocational school or some college	242 (94.6)	15 (5.4)	0.1, (4.1 to 4.6)	236 (91.7)	21 (8.3)	3.1 (7.1 to 1.5)
	High school or less	279 (90.8)	39 (9.2)	3.9 (10 to 2)	272 (88.2)	47 (11.8)	6.6 (13.4 to 1.7)
**Preferred language for SMS text messages**							
	English	1270 (94)	84 (6)	Reference	1254 (92.6)	100 (7.4)	Reference
	Spanish	61 (86.7)	20 (13.3)	7.2 (27.1 to 1.8)	59 (79.8)	22 (20.2)	12.7 (27.2 to 2.8)

**Table 3:** Relationship between demographic factors and caregiver-reported experiences with receiving an SMS text message from a doctor or their office.

		Has received texts from doctors’ office		
		Adjusted, n (%)		Risk difference (95% CI)
		Yes	No	
**Child ethnicity**				
	Not Hispanic or Latino	71.5 (855)	28.5 (323)	Reference
	Hispanic or Latino	74.2 (175)	25.8 (80)	2.7 (4.8 to 10.4)
**Child race**				
	White	72.4 (660)	27.6 (240)	Reference
	Black	75.6 (206)	24.4 (68)	3.1 (5.8 to 11.1)
	Asian	61.1 (79)	38.9 (44)	11.3 (23.6 to 2.3)
	Other	69.6 (60)	30.4 (37)	2.8 (16.5 to 13.7)
**Child age**				
	6-23 months	72.6 (983)	27.4 (368)	Reference
	2-8 years	60.1 (47)	39.9 (35)	12.6 (25.7 to 2.4)
**Child health**				
	Excellent	72.2 (709)	27.8 (270)	Reference
	Very good	72.2 (255)	27.8 (97)	0.04 (6.3 to 7.1)
	Good, fair, or poor	68.6 (61)	31.4 (36)	3.6 (12.8 to 8.4)
**Child insurance type**				
	Commercial insurance	71 (660)	29 (241)	Reference
	Public insurance or uninsured	73.5 (369)	26.5 (162)	2.5 (6.8 to 9.9)
**Caregiver relation to child**				
	Mother	73.5 (933)	26.5 (341)	Reference
	Father, grandparent, or other	58.8 (96)	41.2 (62)	14.7 (23.7 to 5.9)
**Caregiver age**				
	<30 years	67.8 (328)	32.2 (154)	Reference
	30-34 years	72.9 (367)	27.1 (134)	5.2 (0.8 to 11.6)
	35-39 years	74.3 (226)	25.7 (79)	6.6 (2.7 to 18.2)
	>40 years	79.2 (80)	20.8 (26)	11.4 (0.2 to 21.2)
**Caregiver education**				
	Masters or doctorate	77.7 (260)	22.3 (76)	Reference
	Associates or bachelors	71.2 (375)	28.8 (144)	6.5 (11.8 to 0.6)
	Vocational school or some college	72 (184)	28 (73)	5.8 (13.2 to 2.1)
	High school or less	66.7 (210)	33.3 (109)	11 (19.6 to 2.3)
**Preferred language for SMS text messages**				
	English	72.8 (985)	27.2 (366)	Reference
	Spanish	53.6 (45)	46.4 (37)	19.2 (30.4 to 4.4)

## Discussion

### Overview

In this study, most but not all participants used texting frequently and had an unlimited SMS text messaging plan; there was also heterogeneity in SMS text messaging usage and plan type. Answering our initial study question, we found that those who texted less than daily and held limited texting plans were still enrolled to receive SMS text message reminders in a pediatric primary care setting. This confirmed our hypothesis that differences in plans and usage of SMS text messaging services would not preclude use observed of SMS text message services. Further, most participants, including those without unlimited plans and who texted less frequently, had past experience receiving SMS text messages from a doctor’s office as part of routine clinical care.

Among available mHealth technologies, SMS text messages have been shown to be an effective means of heath communication [22,27], while also being accessible and easy to use. Although our study demonstrates participants’ willingness to engage with a mHealth SMS text message intervention even with limited SMS text messaging plans, accommodating families with limited SMS text messaging plans might include asking families what types of SMS text messages they prefer to receive and limiting messages to those deemed highest value. Although there are disparities in smartphone service coverage in the United States, SMS text message reminders can be transmitted to basic cellular devices with use >90% across all age, racial, ethnic, regional, educational, and income groups in the United States. Lower-income caregivers may lose access to or change phone numbers; however, challenges in reaching this group could be mitigated with updates to patient information at each patient encounter [28].

Prior research indicates that caregivers may prefer SMS text message reminders for immunizations over other forms of reminders and perceive more benefits than barriers, such as prohibitive costs or privacy issues, with SMS text message communication [14,27]. However, these studies are not recent [14,28,29,30]. This study adds to the literature by providing new information about prior SMS text messaging experiences by a convenience sample of caregivers, even among those with differing texting plans and patterns of use. This is important as SMS text messages are becoming a more used mode of communication in pediatric primary care settings.

There are several limitations to this study. All participants were enrolled in a trial involving SMS text message reminders. We do not have information regarding texting patterns from families who did not choose to participate. Although this study involved a convenience sample of heterogeneous participants in many states, it is not nationally representative, and results do not reflect the attitudes of all caregivers. Despite these limitations, the study sample was large, and the survey response rate was high (69%).

The relative widespread SMS text message use of caregivers who enrolled in this SMS text message study suggests that SMS text message communications in a primary care setting are acceptable to caregivers with a diverse range of SMS text messaging patterns and plan type. These findings support the continued development of SMS text messaging interventions in primary care.

### Conclusions

Infrequent texting and lack of access to an unlimited SMS text messaging plan did not preclude family enrollment in influenza vaccine reminders in a pediatric primary care study.

## Abbreviations

AAP: American Academy of Pediatrics
CHOP: Children’s Hospital of Philadelphia
HHS: Health and Human Services
HRSA: Health Resources and Services Administration
NICHD: National Institute of Child Health and Health Development
NIH: National Institutes of Health
PROS: Pediatric Research in Office Settings

## Data Availability

The data sets generated and analyzed during this study are available from the corresponding author on reasonable request.
